# A proposed framework for point of care lung ultrasound by respiratory physiotherapists: scope of practice, education and governance

**DOI:** 10.1186/s13089-022-00266-6

**Published:** 2022-06-16

**Authors:** Mike Smith, Simon Hayward, Sue Innes

**Affiliations:** 1 School of Healthcare Sciences, College of Biomedical and Life Sciences, Cardiff University, Cardiff, UK; 2 Lung Ultrasound, Blackpool Teaching Hospitals NHS Foundation Trust, Blackpool, UK; 3 School of Sport, Rehabilitation and Exercise Sciences, University of Essex, Colchester, UK

**Keywords:** Point of care ultrasound, PoCUS, Lung ultrasound, Physiotherapy, Physical therapy, Respiratory therapy, Framework, Scope of practice, Education and competency, Capability, Governance

## Abstract

**Background:**

Point of care ultrasound (PoCUS) has the potential to provide a step change in the management of patients across a range of healthcare settings. Increasingly, healthcare practitioners who are not medical doctors are incorporating PoCUS into their clinical practice. However, the professional, educational and regulatory environment in which this occurs is poorly developed, leaving clinicians, managers and patients at risk.

**Main body:**

Drawing upon existing medical and non-medical literature, the authors present a proposed framework for the use of PoCUS. Throughout, mechanisms for applying the principles to other professionals and healthcare settings are signposted. Application of the framework is illustrated via one such group of healthcare practitioners and in a particular healthcare setting: respiratory physiotherapists in the UK.

In defining the point of care LUS scope of practice we detail what structures are imaged, differentials reported upon and clinical decisions informed by their imaging. This is used to outline the educational and competency requirements for respiratory physiotherapists to safely and effectively use the modality. Together, these are aligned with the regulatory (professional, legal and insurance) arrangements for this professional group in the UK.

In so doing, a comprehensive approach for respiratory physiotherapists to consolidate and expand their use of point of care LUS is presented. This provides clarity for clinicians as to the boundaries of their practice and how to train in the modality; it supports educators with the design of courses and alignment of competency assessments; it supports managers with the staffing of existing and new care pathways. Ultimately it provides greater accessibility for patients to safe and effective point of care lung ultrasound.

For clinicians who are not respiratory physiotherapists and/or are not based in the UK, the framework can be adapted to other professional groups using point of care LUS as well as other point of care ultrasound (PoCUS) applications, thereby providing a comprehensive and sustainable foundation for PoCUS consolidation and expansion.

**Conclusion:**

This paper presents a comprehensive framework to support the use of point of care LUS by respiratory physiotherapists in the UK. Mechanisms to adapt the model to support a wide range of other PoCUS users are outlined.

## Background

The use of ultrasound imaging outside of traditional radiology settings is an area of rapid growth [1]. Point of care ultrasound (PoCUS) can be regarded as the immediate or concurrent integration of ultrasound imaging into delivery of care or decision making as part of patient management [2, 3]. Increasingly this is used by healthcare practitioners who are not medical doctors.

Ultrasound imaging is a modality that requires high levels of skill and experience to use and interpret. Furthermore, the expansion in use of ultrasound imaging by healthcare professionals without a formal background in medical imaging can raise concerns, including quality assurance, missed or mis-diagnosis and litigation [4–6]. Mechanisms to address such concerns are therefore required.

In this paper, the authors outline a framework approach they have developed which is designed to support the consolidation and expansion of PoCUS. Alongside prompts for how other PoCUS groups could apply the framework approach, its application is illustrated using the example of physiotherapists in the UK who specialise in respiratory care.

Respiratory care is an area of clinical practice where PoCUS has a potentially valuable role to play for patients with respiratory impairment, including those in community/primary care and secondary care (including critical care) settings [7, 8]. Physiotherapists who specialise in respiratory care have a crucial role to play in the pathway for many such patients [9, 10]. With point of care lung ultrasound (LUS) being increasingly performed by respiratory physiotherapists to investigate the pleura and lung parenchyma [11, 12], a framework to support these clinicians, the wider care pathway and ensure patient safety is necessary. Existing literature in the area of barriers and facilitators to the use of point of care LUS was drawn upon in order to inform the mechanisms presented in this paper, thus framing them in light of existing work.

The objectives of the paper are to (i) describe the principles and component parts of a proposed framework approach for PoCUS, (ii) illustrate these through the example of physiotherapists in the UK using point of care LUS and (iii) provide prompts for other PoCUS users to consider how they could apply the framework approach.

## Main text

### A proposed framework approach to supporting PoCUS

Ultrasound imaging has the potential to transform the clinical effectiveness and efficiency of countless healthcare components and pathways, by placing real time imaging into the hands of triaging and treating clinicians. However, the sheer number of potential tissue types, organ systems, disease processes and clinical differentials that can be encountered when imaging means that mechanisms to frame PoCUS activity in order to provide quality assurance are essential. Furthermore, the expertise of imaging professionals (such as radiologists and sonographers), combined with considerations of education, competency and governance, means that PoCUS must be contextualised accordingly.

**Fig. 1:**
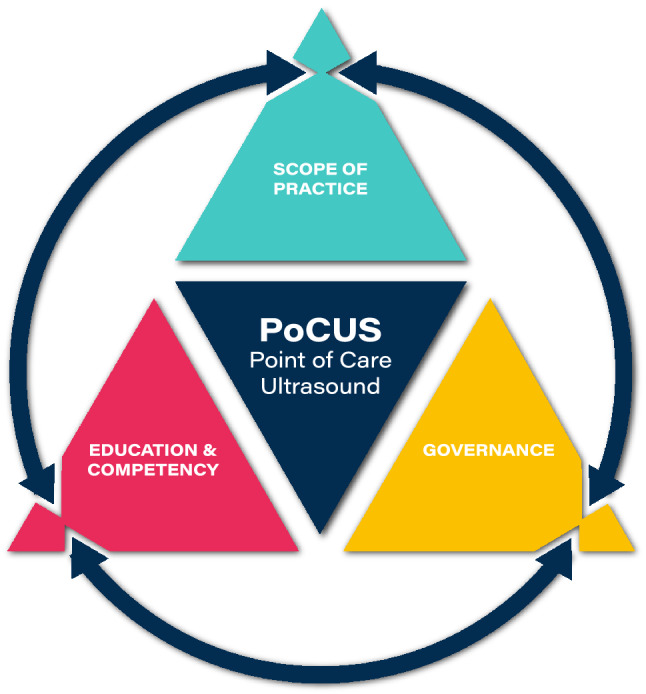
PoCUS framework triangle. Concept by Dr Mike Smith (Cardiff University, UK), created by Dan Molloy (freshwater.media), copyright 2021 Dr Mike Smith

Recognising the above, the authors propose a framework for PoCUS (Fig. 1), comprising the elements of (i) scope of practice (ScoP), (ii) education and competency and (iii) governance. These terms are well established in the published literature, having been described by authors, such as Ambasta et al. [13], LoPresti et al. [14], Lee and DeCara [15] and Teunissen et al. [16]. The framework concept is that each of the elements inform and must be in alignment with each other in order for robust delivery of PoCUS. In the same way, new areas of PoCUS activity can be established by developing or resolving one or more of the elements, thereby ensuring alignment across the framework. Figure 1 provides a visual representation of the framework approach. At the time of writing, the PoCUS framework approach is being applied in a range of other areas of clinical practice; as such this manuscript shares some generic content with the publication Smith et al. [17].

Whilst this paper draws upon the example of point of care LUS by respiratory physiotherapists in the UK to illustrate the application of these principles, at the start of each section, prompts are presented to support other healthcare professionals and those working in different healthcare systems with how the framework approach might be used.

#### Respiratory physiotherapy

Prompts for other PoCUS users regarding the context for their clinical activities:

What is the generic (i.e. non-PoCUS) nature of the professional group and healthcare setting?

Considerations include the following:

- Professional regulation and autonomy;
- The clinical role(s) they undertake and the care pathway(s) they work within.

In the United Kingdom (UK), respiratory physiotherapists are autonomous clinicians who hold a formal qualification as a physiotherapist. Typically this will be a minimum of a Bachelor of Science with Honours (BSc(Hons)) Physiotherapy degree or post-graduate, pre-registration equivalent (e.g. Master of Science (MSc) Physiotherapy Pre-Reg). Combined with their registration with the Health and Care Professions Council (HCPC) they can use the protected title of ‘Physiotherapist’ and are eligible to join the professional body the Chartered Society of Physiotherapy (CSP) [18].

Respiratory physiotherapists use a range of assessment, monitoring and treatment approaches as part of the multi-disciplinary management of patients with respiratory impairment. This includes subjective and clinical history taking along with a combination of assessment procedures they undertake (such as auscultation, strength testing and mobility assessment) and assessment procedures that may have been undertaken by other members of the multi-disciplinary team (e.g. chest imaging, arterial blood gases (ABG’s), etc.), as part of their clinical examination. Applying a combination of clinical reasoning and patient-centred care, they independently formulate and apply treatment approaches, such as chest clearance techniques, lung volume recruitment, strategies to reduce work of breathing, optimising of gaseous exchange, whole body mobility and patient education [19–21].

Respiratory physiotherapists may be involved in management of long-term respiratory conditions in the community/primary care settings [9, 22]. In secondary care settings they also have a role in the management of acute respiratory impairment or exacerbations which may be secondary to trauma, surgery or wider/multi organ disease [10, 23, 24]. They also have a vital role to play in critical care environments, working alongside intensivists, anaesthetists and other members of the critical care team. In this regard, there is a degree of overlap with that of nurses or allied health professionals in advanced critical care practitioner roles. Furthermore, their work across wider healthcare settings overlaps with ‘physical therapists’ working in respiratory areas and professionals termed ‘respiratory therapists’ in the United States of America and other similar healthcare systems. Many of the point of care LUS framework elements described in this paper could therefore directly apply to these other professional groups.

### A proposed framework for point of care LUS by respiratory physiotherapists

#### Permitted use of ultrasound imaging in the UK by physiotherapists

Prompts for other PoCUS users: for the profession (in the specific healthcare setting of interest), what is the current situation relating to their use of ultrasound imaging?

Potential permutations include the following:

- Use of ultrasound imaging is permitted from a medicolegal and/or professional autonomy and permission perspective; or it is not.
- Use of ultrasound imaging is accepted by other members of the care pathway (including career imaging, such as radiology or sonography); or it is not.
- Ultrasound imaging is permitted/accepted only for observation of structures (non-diagnostic); or its use for sonographic diagnosis is also permitted.

The above provides the foundation for framing the consolidation ± expansion of the existing permissions via the subsequent ScoP, education and competency and governance elements.

The CSP, as the UK professional body for Physiotherapy, uses 4 pillars of practice [25] to describe the fundamentals of the scope of practice of the physiotherapy profession; one of these pillars is ‘therapeutic and diagnostic technologies’. Ultrasound imaging and its application as a diagnostic modality aligns with this pillar. As such, the standards of proficiency for physiotherapists (as set out by the HCPC [26]) apply to PoCUS (including point of care LUS)—just as they would for any other area of permissible physiotherapy practice. In terms of the wider UK health system, ultrasound imaging is an unregulated modality and at the time of writing there is no protection of title for ‘Sonographer’.

Over the last decade or so there has been a sustained and rapid increase in the number of physiotherapists in the UK using ultrasound imaging, predominantly in a PoCUS capacity. This is across a wide range of clinical specialities (musculoskeletal (including rheumatology), pelvic health, image-guided injections (including in management of spasticity for patients with neurological disorders)) and settings (both research and healthcare settings: community/primary care and secondary care—including First Contact Practitioner and Advanced Clinical Practice roles).

ScoP related to ultrasound imaging refers to numerous elements, including the tissues to be imaged, the clinical and sonographic differentials and the subsequent clinical decision making. These are largely framed by governance considerations relevant to physiotherapists in the UK who are members of the CSP with the relevant level of CSP provided indemnity insurance. As such, the ScoP must demonstrably align with their role as a physiotherapist [27] in order for them to be using it in their capacity as a physiotherapist, and for CSP professional liability insurance (PLI) coverage to apply. The explicit alignment of PoCUS imaging with the scope of clinical practice of the professional has been previously described relating to the use of LUS during the COVID-19 pandemic [28].

Those elements deemed within ScoP should be discrete from tissues imaged, clinical & sonographic differentials and subsequent clinical decision making that is outside of a physiotherapist’s ScoP. This provides a mechanism to address potential physiotherapy management concerns around litigation risk, as identified by Hayward et al. [29]. An emphasis upon the interpretation and subsequent clinical decision making around the imaging also aligns with the (I-AIM) Indication, Acquisition, Interpretation, Medical Decision-making framework for point of care LUS described by Kruisselbrink et al. [30].

#### ScoP: clinical and sonographic

Prompts for other PoCUS users regarding ScoP considerations:

- What are the high value areas of existing clinical practice that would potentially be enhanced (e.g. safer, expedited, more accurate, shorter care pathway, etc.) by the integration of PoCUS?
- Framed by the earlier considerations (i.e. for the profession, in the specific healthcare setting of interest, what is the current situation relating to their use of ultrasound imaging), what tissues are to be imaged, clinical & sonographic differentials to be derived (if any) and subsequent clinical decision making to be performed?
- By default, what tissues imaged, clinical & sonographic differentials and subsequent clinical decision making are not to be performed.

Table 1 provides an indicative list of imaging that may be performed and how this is used pertaining to point of care LUS by respiratory physiotherapists. These mirror the importance of high clinical value applications of point of care LUS as identified by Hayward et al. [29] and builds upon proposals from Leech et al. [31] and LeNeindre et al. [32] alongside consensus work by Volpicelli et al. [33]. In the above regard a ‘rule in’ approach is emphasised whereby clinical assessment and reasoning formulate a priori the likely differentials—which ultrasound imaging is then used to identify (as appropriate) [34]. This imaging process-driven hypothesis testing is in contrast to a ‘rule out’ approach more typically employed by imaging services provided by imaging professionals, such as radiologists and sonographers. In those cases, a range of potential sonographic findings (and subsequent clinical differentials) may be ruled out via the imaging [35]. Also of note here is the specific benefit of PoCUS LUS performed by the respiratory physiotherapist as a tool to ‘monitor severity and response to treatment/intervention’; this is arguably distinct from a ‘rule in’/‘rule out’ approach. This example of imaging being integrated into the management of their patient, further emphasises the value of a treating clinician using PoCUS.

**Table 1:** Indicative imaging performed and how this information is used pertaining to point of care LUS by respiratory physiotherapists

^a^Indicative imaging performed	Role of the imaging of these structures	Role within physiotherapy and wider MDT patient management
^∆^Recognition of normal thoracic structures and adjacent organs as landmarks • Subcutaneous tissues, ribs, pleura and diaphragm • Heart, liver, spleen and kidneys • Aorta and vena cava	Awareness of spectrum of ‘normal’ presentations. Landmark identification serves as mechanism to enhance accuracy of imaging; integral aspect of protocol-based imaging	Recognition of ‘normal’ as part of sonographic and clinical differential diagnosis process. Standardised approach to imaging as quality assurance mechanism
^◊^Identification of ultrasound appearances of normal aerated lung including • Pleural line and lung sliding (in 2D/B mode and M mode) • Normal aerated lung (including A-line and B-line artefacts)	Awareness of ‘normal’ presentations	Recognition of ‘normal’ as part of sonographic and clinical differential diagnosis process
Recognition of pleural fluid: • Appearances of pleural fluid and pleural thickening • Estimation of pleural effusion volume • Demonstration of sinusoid sign on M mode • Distinguishing between pleural and abdominal fluid collection	Building upon ^∆^ and ^◊^, sonographic differential diagnosis, description and (where appropriate) estimation of pleural effusion	Feeds into clinical differential diagnosis process; also monitoring of severity and response to treatment/intervention
Recognition of consolidation/atelectasis: • Ultrasound appearances of consolidated/atelectatic lung • Ultrasound appearances of air and fluid bronchograms	Building upon ^∆^ and ^◊^, sonographic differential diagnosis and description of consolidation/atelectasis and types of bronchograms	Feeds into clinical differential diagnosis process; also monitoring of severity and response to treatment/intervention
Recognition of interstitial syndrome: • Recognition of B-lines • Differentiating between normal and pathological B-lines	Building upon ^∆^ and ^◊^, sonographic differential diagnosis and description of interstitial syndrome	Feeds into clinical differential diagnosis process; also monitoring of severity and response to treatment
Use of ultrasound to exclude pneumothorax: • Recognition of signs of pneumothorax (B mode and M mode) • Absence of lung sliding, B-lines and lung pulse • Presence of lung point	Building upon ^∆^ and ^◊^, exclusion of pneumothorax	Feeds into clinical differential diagnosis process

^a^This column draws upon the focused ultrasound intensive care (FUSIC) Lung Ultrasound accreditation written by Dr Ashley Miller (Co-Chair FUSIC Committee; Consultant Intensivist Shrewsbury Telford Hospital); https://www.ics.ac.uk/Society/Learning/FUSIC_Accreditation

The presence and nature of pleural effusion, consolidation/atelectasis, interstitial syndrome and pneumothorax identified via ultrasound imaging may be commented upon by respiratory physiotherapists. Monitoring of these presentations in relation to patient management (e.g. patient positioning, manual techniques, recruitment strategies, pharmaceutical and exercise management) may also be commented upon. In all cases the sonographic findings would be commented upon in the context of other respiratory physiotherapy assessment procedures (e.g. clinical assessment, chest radiograph, auscultation, ABG’s, etc.). It is noted that primary diagnosis of pathologies based solely upon ultrasound imaging would be outside the ScoP remit for respiratory physiotherapists; this differentiates the sonographic ScoP from that of a radiologist or sonographer for whom primary identification of lung pathologies or differential diagnosing of pathologies based solely upon ultrasound imaging would be a core activity.

In describing the point of care LUS scope of practice for respiratory physiotherapists, this (by a process of elimination) also clarifies what tissues imaged, clinical & sonographic differentials and subsequent clinical decision making is not to be performed. Indicative examples of imaging outside the respiratory physiotherapist’s ScoP would include the following:

- Cardiac and vascular pathology, including abdominal aortic aneurysm (AAA), cardiac tamponade, echo-cardiogram, inferior vena cava (IVC) or mediastinum.
- Suspicious mass, including any benign or metastatic masses
- Other abnormalities of the cardio-thoracic system, including fluid status
- Other abnormalities of the thoracoabdominal region, including liver, spleen, kidney or gastric pathologies; causes of free abdominal fluid i.e. ascites or blood

Whilst the above lie outside of scope of practice, they may be identified as either incidental or concurrent imaging findings. Just as a physiotherapist has a duty of care to escalate patient elements that may be strictly out of remit, such as evidence of abuse or risk of self-harm, it is also necessary that they can act upon any imaging concerns [35]. In this regard a clear protocol must be in place for the clinician to be able to discuss concerns and for the clinical assessment and/or imaging of the patient to be escalated; this includes where pathologies/presentations (that are within ScoP; as per Table 1) are identified for the first time in that episode of care—by the physiotherapist using point of care LUS. This could potentially include options for direct communication with those who have access to more specialist US imaging expertise, other imaging modalities and/or surgical or medical opinion. This highlights the importance of respiratory physiotherapists undertaking their ultrasound imaging as part of a wider clinical and imaging team.

The comprehensive defining of ScoP—as a foundation for targeted educational provision (see next section)—helps address one of the frequently cited barriers to uptake of PoCUS (including point of care LUS), namely the required significant investment of clinician time to learn and perform point of care LUS [36, 37].

#### Education and competency

Prompts for other PoCUS users regarding education and competency considerations:

- What are the existing routes for undertaking education (and demonstrating competency) that align with the above ScoP? If no bespoke solutions exist, where can shared learning be undertaken with specialities (including career imaging)?
- Where should the PoCUS skills sit within a pre-qualification/post-qualification/career-progression timescale?

As per Fig. 1, the education and competency elements must align with and should be reflective of the ScoP. In this regard a description of the respiratory physiotherapy-specific components are outside of the remit of this paper, but would include both formal and informal ‘work place based’ training, mentoring and feedback regarding pathology, clinical reasoning and clinical management. It is noted that the term ‘capability’ is increasingly used in the healthcare literature, whilst ‘competency’ is typically used in the ultrasound literature; for the purposes of this framework the terms are essentially used interchangeably.

With regard to ultrasound imaging-specific education and competency, two primary routes exist for PoCUS education (which is orientated towards patients with respiratory impairment) in the UK: (i) via a Higher Education Institution (HEI) course and (ii) via professional societies. Examples of the latter include the Intensive Care Society (with Focused Ultrasound for Intensive Care, FUSIC) and the Society of Acute Medicine (with Focused Acute Medicine Ultrasound, FAMUS). In practical terms, with HEIs these are often ‘negotiated module(s)’ within a broader Post-graduate Certificate (PgC) or Masters qualification in medical imaging, whilst professional societies are typically based around clinical competency assessments alongside routine clinical practice and training. It is recognised that due to various factors (including a lack of regulation around the title ‘sonographer’) absolute requirements regarding competency assessment are not present and as such, the likelihood is that both routes will continue to flourish in the UK.

Key considerations for course providers, individual learners and their managers include the following: whether the full range of foundation and speciality-specific elements are taught and assessed (see next paragraph); whether the course has been externally scrutinised by a body, such as the Consortium for the Accreditation of Sonographic Education (CASE), and the importance of demonstrable competency via a formal assessment route [38] in terms of any subsequent need to defend the clinical practice of an individual.

Relating to the above, Table 2 provides a summary of key considerations regarding post-registration (see next paragraph) education and competency, both generically for ultrasound imaging and specifically for respiratory physiotherapists; this aligns with the UK’s performance, interpretation and reporting on ultrasound examinations National Occupational Standard [39]. This builds upon the work of Cholley et al. [40] who were unable to report on a clear consensus around the number of scans performed or cases seen, but did recommend theoretical and practical ultrasound education aiming for clinician competency [40]. The systematic review on LUS education by Pietersen et al. [41] reported there was no clear evidence on which methods were optimal for either the teaching LUS or the summative assessment [41]. As such, the authors of this paper have drawn upon their extensive background in PoCUS education and competency (including for other areas of Physiotherapy and non-Physiotherapy PoCUS education provision: through longstanding involvement with CASE) in proposing formats of teaching, formative and summative assessment. When combined with Table 1, these essentially provide a template for a potential point of care lung ultrasound curriculum. Conversely, if an individual were to undertake a pre-existing course then, mapping across to the content in Tables 1 and 2 would enable interested parties to determine whether the requisite education and competency components are addressed. The purposeful alignment of ScoP with education and competency components provides a mechanism to address the often levelled criticism that more uniformity and higher standards are required in PoCUS training, including in LUS [31, 41].

**Table 2:** Key considerations regarding education and competency

Educational elements	Potential educational mechanisms and *assessment of competency*	Relevance to scope of practice
1. Critical understanding of how an ultrasound image is generated. Includes • Fundamental physics as applied to ultrasound • Artefacts and how to manage/interpret them	Face to face teaching and/or provision of online/pre-reading material *Multiple choice questions/coursework around imaging scenario(s)*	As core underpinning principles, PoCUS users require an awareness of the limitations of the modality and how to interpret the sonographic representation of tissues
2. Image optimisation. Includes: • The function of ultrasound machine settings (relating back to fundamental physics principles) • ‘Knobology’ and application of image optimisations strategies in practical scenarios	Include provision of online/pre-reading material. However, hands on teaching is essential—for example, using phantoms, simulators, healthy subjects *Overlap with 1. However hands on assessment is essential and could be integrated with objective structured clinical examination (OSCE) format*	Image optimisation techniques are essential for high quality imaging practice and allows for adaptation to different ultrasound machines and clinical scenarios
3. Safety and professional considerations. Includes: • Thermal and non-thermal effects; ALARA (As Low As Reasonably Achievable) principles • Infection prevention and control • Use of evidence based protocols; taking and labelling of standardised views • Reporting terminology • Secure storage of images and their integration into the electronic patient record of the wider care pathway • Awareness of benefits and limitations of ultrasound imaging and awareness of role of other imaging modalities • Indications for performing a scan; includes informed patient consent	Include provision of online/pre-reading material, although practical teaching is essential *Overlap with 1 and 2. Hands on assessment is essential and could be integrated with OSCE format*	Safety considerations that are generic in ultrasound imaging and specific to respiratory system scanning Standardised image taking, recording and reporting allow for consistency with other ultrasound imagers As professionals without a pre-existing foundation in imaging, awareness of the indications for, and the role of imaging modalities is essential
4. Imaging of ‘normal’ anatomy. Includes • Ability to use standardised protocols, recognise normal anatomical variation and adapt imaging based upon factors such as high levels of adipose tissue, poor patient positioning or poorly imaging tissues	Include provision of online/pre-reading material. However, hands on teaching is essential—using simulators and more importantly healthy subjects. Requires a range of ‘normal’ presentations *Overlap with 1 and 2. However hands on assessment is essential and could be integrated with OSCE format*	Awareness of the range of ‘normal’ presentations provides a reference for identifying deviations from normal Provides an opportunity to familiarise self with strategies for addressing sub-optimal imaging prior to moving onto imaging ‘non-normal’
5. Imaging of ‘non-normal’ anatomy. Includes • Awareness of the range of sonographic presentations associated with different pathologies/clinical scenarios. Where applicable, how to perform a differential sonographic diagnosis • How to adapt imaging based upon factors, such as high BMI, poor patient positioning or poorly imaging tissues • Clinical relevance (or otherwise) of sonographic findings, including false + ve/–ve	Include provision of online/pre-reading material. However, hands on teaching is essential—using simulators and more importantly patients. Requires a range of different pathologies/clinical presentations Essential requirements include availability of suitably qualified and experienced mentor, access to an appropriate patient mix and directly supervised scanning *Overlap with 1 and 2. However hands on assessment is essential. Directly supervised assessment of scanning patients is the recommended assessment approach along with logbook of scans undertaken and critical reflection upon subsequent clinical decision making*	Awareness of the range of pathological / clinical presentations, including spectrum of severity. Ability to adapt imaging practice to address sub-optimal imaging An awareness of how to interpret the imaging findings, implement them into clinical decision making/treatment—and communicate them to the other care pathway members (as appropriate)

Due to the necessity for high level clinical reasoning skills (required to appropriately choose to use ultrasound imaging and to integrate those findings into patient management) a physiotherapist undertaking point of care LUS requires a substantial level of respiratory clinical skills and experience. As such, training in point of care LUS should occur at post-graduate level and by someone with the appropriate level of experience in respiratory care (which is relevant to their subsequent point of care LUS ScoP).

#### Governance

Prompts for other PoCUS users regarding governance considerations:

- These are substantially framed by the earlier considerations “for the profession, in the specific healthcare setting of interest, what is the current situation relating to their use of ultrasound imaging”; however, consideration of progressive mechanisms to address constrictions on practice should be considered. Where are there other precedents for progress?
- Medicolegally: consider use of terminology to explicitly clarify the nature of the scan (see section ‘Governance’)
- Quality assurance mechanisms must be robustly and proactively addressed, in order to consolidate (and particularly in the advancement of) ScoP.

As noted earlier, insurance considerations and professional regulation substantially influence the ScoP for respiratory physiotherapists performing point of care LUS in the UK. In the same way, however, the defining of ScoP confers numerous governance and care pathway benefits, as outlined in Table 3. This includes awareness by other care pathway members of what the scan is and is not undertaken for and support for clinical managers in care pathway design and staffing.

**Table 3:** Governance and care pathway benefits of describing scope of sonographic and clinical practice

‘Audience’	Utility
The referring clinician and other members of the care pathway (e.g. intensivist, respiratory physician, etc.)	The referring clinician is aware of what the physiotherapist has the remit to scan and what can be inferred from the scan. Just as importantly they are aware of the limitations of the scan and that for aspects that are out of scope of practice (e.g. imaging for or identification of cardiac pathology, causes of free abdominal fluid, etc.) that the scan is not for the purposes of either confirming or excluding
Patient	In providing informed consent (where applicable), the patient is aware of what the imaging is being performed for, but just as importantly what the imaging is not being performed for (as above)
Professional body and regulatory body	The professional and regulatory bodies can identify that the imaging being performed and the clinical inferences derived from the scan are permissible for that clinician/profession, and correspondingly can confer permission to proceed/professional indemnity coverage
The insurer (professional body, employer or 3rd party)	The insurer can consider the scope of sonographic and clinical practice to determine whether insurance coverage can be provided and to more accurately determine any insurance premium
The manager of the clinician	Provides clarity regarding what the clinician will be imaging and what they will be doing with that information. As such, allows for the design and staffing of existing and new care pathways
The education provider	Provides clarity regarding the requisite education content and the necessary areas for evidencing competency. This includes the clinical indication for and the clinical implementation of the sonographic information
The clinician	The clinician can undertake the necessary education and competency assessment requirements, and can be confident that the relevant governance elements have been addressed and that clinicians upstream/downstream are aware of the remit of the scan

The use of terminology to explicitly clarify the nature of the scan is encouraged. An example of the professional context to the imaging process that could be communicated to colleagues is as follows: “Aligning with the scope of clinical and sonographic practice outlined in **this publication** for respiratory physiotherapists performing point of care lung ultrasound in the UK, this ultrasound scan is undertaken for the purposes of assessing pleural and lung parenchyma pathology as an adjunct to respiratory physiotherapy management. The identification of other anatomical or pathological elements is explicitly beyond the scope of practice of the clinician. Therefore, the scan cannot be relied upon to either confirm or exclude any such anatomical or pathological elements”. Such an approach reflects the benefits identified by Hayward et al. [29] of a shared, clear understanding of scope and remit.

Quality assurance considerations include data protection, storage of images, continuous professional development and access to a second opinion. As PoCUS is often undertaken in non-radiology settings, direct access to Picture archiving and communication system (PACS) for secure storage and backing up of sonographic images may not be immediately available. As highlighted by Wolstenhulme et al. [42] this poses a risk to data security as well as continuity of care and the ability to review image quality. Integration of PoCUS imaging into the electronic patient record and its accessibility by the wider care pathway are critical considerations by which duplication of imaging can be avoided (with the caveat that imaging may be undertaken with different remits), changes over time can be monitored and acceptability by other PoCUS users and career ultrasound imaging professionals can be facilitated. Mechanisms for the secure storage of sonographic images need to be addressed as a priority (including their integration into formal PoCUS education), and this may include bespoke mechanisms to upload to PACS or the use of other secure image storage capacity as advised by a data compliance officer (such as encrypted, cloud-based repositories), and reporting systems which can integrate with pre-existing patient record systems.

As part of best practice, respiratory physiotherapists using ultrasound imaging should undertake ongoing audit of their practice. Double scanning with an experienced colleague and discussion of complex cases with a more experienced imager should also be undertaken as part of continuing professional development and quality assurance activities [43–45]. Collectively, the above governance considerations provide robust mechanisms to address concerns raised by Leech et al. [31] that would otherwise inhibit the widespread implementation of point of care LUS by physiotherapists.

### Broader considerations

#### Expansion of ScoP

Prompts for other PoCUS users regarding ScoP expansion considerations:

- What PoCUS applications (e.g. imaging techniques, target tissues, patient groups, care pathways, procedures, etc.) might conceivably be undertaken?
- Can these be undertaken using existing permissions—or do they require new permissions?
- What existing or potential routes are there for gaining and evidencing competency?

In describing a clinical and sonographic ScoP for physiotherapists in the UK who specialise in respiratory care, this is not intended to stifle innovation or development of respiratory physiotherapy clinical practice or roles. For example, there is the potential for respiratory physiotherapists to undertake structural and functional assessment of the diaphragm as part of their physiotherapy management of patients with respiratory impairment. Applying the principles outlined in this paper means that where the activity demonstrably sits within the physiotherapy management of a patient, professional regulation and CSP insurance considerations would conceivably have already been addressed. Following this, education and demonstrable competency considerations would need to be satisfied along with agreement with clinical managers—at which point such a role could be undertaken.

#### Research

Prompts for other PoCUS users regarding research considerations:

- The evidence base for incorporating PoCUS by a profession in the specific healthcare setting of interest should be scrutinised and added to.
- Evidence can be drawn upon from other professions or healthcare settings, though consideration should be explicitly given to key caveats or limitations.
- Consideration of how POCUS can also be used as a research tool.

Evidence relating to the ability of ultrasound imaging to identify different tissues and disease processes can be drawn from a range of traditional imaging (e.g. radiology and career sonography) and point of care LUS sources (e.g. intensive care, critical care and emergency care medicine). This can in part address concerns raised in Hayward et al. [29] about the limited respiratory physiotherapy-specific evidence base in relation to the application of point of care LUS.

Nonetheless, it is essential to add to the evidence base relating to if, where and how point of care LUS can enhance clinical effectiveness and efficiency of healthcare components and pathways. This includes consideration of optimal education and service delivery models as well as whether the use of imaging may negatively impact clinical outcomes or efficiency of resource use.

In relation to respiratory physiotherapists performing point of care LUS, evidence can be drawn from other professional groups, such as intensivists, anaesthetists and respiratory physicians [33]. Nonetheless, the specifics of how respiratory physiotherapists work need to be reflected. As noted earlier, the degree of overlap with advanced critical care practitioners and ‘respiratory therapists’ provides a potential opportunity for pooled research and inter-professional collaboration.

It should also be noted that PoCUS can also play a valuable role as a research tool or outcome measure. Examples of how respiratory physiotherapists are using of point of care LUS in this capacity can be found in the scoping review by Hayward and Janssen [11]. Larger trials which (at the time of writing) are using point of care LUS as an outcome measure to research physiotherapy practice are underway, including the US-ADEPT trial [46] and the DUCHESS trial by [47]. Mirroring the principles outlined in this paper, individuals using LUS for research purposes would be expected to have undertaken education components and competency assessments which align with their subsequent LUS research ScoP.

### Alignment with the advanced clinical practice agenda

As a progressive area of highly skilled practice, point of care LUS by respiratory physiotherapists would seem to naturally align with the advanced clinical practice agenda [48] in the UK. We advocate though that point of care LUS has the potential to become a routine part of respiratory physiotherapy practice and that as such these clinicians do not *need* to be operating at ‘advanced level’ or above prior to commencing point of care LUS training. Nonetheless, the 4 pillars of advanced practice (Clinical Practice, Leadership and Management, Education and Research) overlap substantially with the expanding role that is the use of point of care LUS by respiratory physiotherapists. As such we encourage point of care LUS adopters to explore how use of this imaging modality can further advance clinical practice and consultant roles.

### Support for the consolidation and expansion of PoCUS use by other professions and healthcare settings

This paper details the situation for physiotherapists in the UK and in this regard, it is noted that the level of autonomy enjoyed is greater than that of physiotherapists/physical therapists in many other countries. It is hoped therefore that the generic mechanisms outlined in this paper will provide a potential direction of travel for physiotherapists/physical therapists in other geographical regions to advance their use of point of care LUS imaging in a comprehensive and sustainable manner.

As outlined throughout this paper, the PoCUS principles can be adapted to apply regardless of the profession (e.g. medic/physician, nurse, allied health professional, paramedic, etc.) or healthcare setting. However, each profession or healthcare setting may require nuanced solutions due to the intricacies of specific professional autonomy, insurance/regulatory arrangements, accepted practice, etc. In this regard the authors are happy to be contacted by different professions or bodies to support with developing bespoke solutions.

## Conclusions

This paper has proposed a novel framework approach to supporting PoCUS and has applied it for respiratory physiotherapists in the UK using point of care LUS. For the first time this provides this professional group with a comprehensive approach to their use of point of care LUS. As part of this, the insurance considerations and professional regulations substantially influence the ScoP—which encompasses a broad range of imaging elements relating to the physiotherapeutic management of patients with respiratory impairment.

Education and competency assessment considerations are explicitly aligned with the clinical and sonographic ScoP and provide the foundation for robustly addressing a range of governance requirements. These also include elements, such as data security and continuing professional development.

Looking beyond the use of point of care LUS by respiratory physiotherapists, we propose that the framework could provide an adaptable model for supporting the consolidation and expansion of PoCUS across a range of clinical specialities, professions and healthcare settings.

## Data Availability

Not applicable.

## Abbreviations

PoCUS: Point of care ultrasound
LUS: Lung ultrasound
UK: United Kingdom
CSP: Chartered society of physiotherapy
ABG’s: Arterial blood gases
ScoP: Scope of practice
I-AIM: Indication, acquisition, interpretation, medical decision-making
AAA: Abdominal aortic aneurysm
IVC: Inferior vena cava
FUSIC: Focused ultrasound for intensive care
FAMUS: Focused acute medicine ultrasound
CACTUS: Children’s acute ultrasound training
PgC: Post-graduate certificate
CASE: Consortium for the accreditation of sonographic education
